# Evolution of a novel technology for gastroesophageal reflux disease: a safety perspective of magnetic sphincter augmentation

**DOI:** 10.1093/dote/doab036

**Published:** 2021-06-11

**Authors:** Janet DeMarchi, Michael Schwiers, Mark Soberman, Allison Tokarski

**Affiliations:** Medical Affairs, Ethicon Incorporated, Cincinnati, OH, USA; Biostatistics, Ethicon Incorporated, Cincinnati, OH, USA; Medical Safety, Ethicon Incorporated, Cincinnati, OH, USA; Clinical Affairs, Ethicon Incorporated, Cincinnati, OH, USA

**Keywords:** gastroesophageal reflux disease, GERD, LINX, magnetic sphincter augmentation, MSA

## Abstract

Magnetic sphincter augmentation using the LINX® device is a minimally invasive surgical option for patients with gastroesophageal reflux disease. An estimated 30,000 devices have been implanted worldwide. Device removals and erosion are identified risks. The objective of this analysis is to explore the procedure evolution with an emphasis on the removals and associated characteristics that may guide future clinical practice. The Manufacturer and User Facility Device Experience and Ethicon’s complaint databases were queried for all surgical device explants since January 2013. Device unit sales were used to determine the rates. The endpoint was based upon the time from implant to explant. Explant and erosion rates were calculated at yearly intervals and the Kaplan-Meier estimator was used to measure the time to explant. Chi-square analyses were used to investigate the risk of explant associated with the size, geography and implant year. Overall, 7-year cumulative risk of removal was 4.81% (95% Confidence Interval (CI) CI: 4.31–5.36%). The likelihood of removal was significantly related to the device size (*P* < 0.0001), with smaller sizes being more likely to be explanted. The primary reasons for device removal and relative percentages were dysphagia/odynophagia (47.9%), persistent gastroesophageal reflux disease (20.5%) and unknown/other (11.2%). Overall, the 7-year cumulative risk of erosion was 0.28% (95% CI: 0.17–0.46%). The average device size increased from 14.2 beads ± 1.0 in 2013 to 15.3 beads ± 1.2 in 2019 (*P* < 0.001). Surgical technique and perioperative management play an important role in the outcomes. Clinical practice changes since magnetic sphincter augmentation has been incorporated into clinical use are associated with improved outcomes and should be further characterized. Smaller device size is associated with increased removal and erosion rates.

## INTRODUCTION

Magnetic Sphincter Augmentation (MSA) with the LINX® device (Ethicon Incorporated, Cincinnati, OH) is a minimally invasive surgical procedure available to patients diagnosed with gastroesophageal reflux disease (GERD), who are seeking an alternative to full-time acid suppression medication. The LINX device consists of a series of titanium beads with magnetic cores that are connected by independent titanium wires to form an annular shape. The attractive force of the magnetic beads is designed to provide additional strength to keep a weak lower esophageal sphincter (LES) closed. During swallowing, the magnetic beads slide away from each other on the independent titanium wire ‘links’ to allow esophageal distention as the bolus passes by. In the time frame of this analysis, the device was available in five sizes in the USA and four sizes outside the USA (OUS). The device size is identified by the number of titanium beads in the device. For example, a model LXMC15 is a LINX device that is 1.5 Tesla (T) magnetic resonance imaging (MRI) compatible, has a clasp closure and contains 15 beads. The proper device size is determined intraoperatively for each patient using a specific sizing tool designed to measure the circumference of the esophagus.

MSA using the LINX device gained CE mark in 2008 and FDA approval in 2012. The performance of the device in these early trials has been documented in several peer-reviewed publications.^1–6^ Today, the device is commercially available in the USA and select countries in Europe, the Middle East, Asia and South America. To date, over 30,000 devices have been distributed and implanted worldwide. The initial devices had no MRI compatibility, which was increased to the ability to withstand a 0.7 T MRI scan in 2013 and up to a 1.5 T MRI scan in early 2015. The initial devices were placed around the esophagus and were secured by tying sutures attached to eyelets on each end of the device. A clasp closure was implemented in early 2013.

In addition to the device design changes, it is notable that the sizing tool, patient selection, surgical technique as well as perioperative care have all evolved over time as surgeons gained more experience with the technology. The current sizing tool is a laparoscopic tool with a flexible, retractable end as opposed to the first generation sizer that resembled the MSA device itself. The original sizing tool was dropped into the abdominal cavity and wrapped around the esophagus to measure the circumference and determine the appropriate device size. Patient selection has been expanded to include patients with hiatal hernias >3 cm, who were not included in the original study population. The surgical technique has moved from minimal dissection of the area around the gastroesophageal junction to full crural dissection and concomitant hiatal hernia repair. Techniques to manage postoperative dysphagia, such as an eating protocol, the optimal time to consider esophageal dilation and/or steroid treatment, have been explored.

**Fig. 1:**
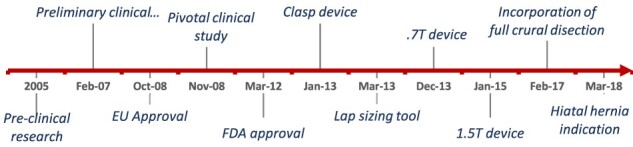
Timeline of procedure and device development

Figure 1 shows the timeline of device and procedure development from preclinical to present day. The following sections outline the evolution that occurred with the sizing tools and technique and the surgical procedure.

The objective of this publication is to describe this multivariate evolution and the safety profile of the currently available device. This publication is based upon internal and external data and has been authored entirely by Ethicon personnel.

## METHODS

The Manufacturer and User Facility Device Experience (MAUDE) database and Ethicon’s complaint database were queried for all device removals. The MAUDE database houses medical device reports submitted to the FDA by mandatory reporters, which include device manufacturers, importers and device user facilities as well as voluntary reporters, such as health-care professionals and patients. These reports include any event that may be a device-related death, injury or malfunction. The reporting period includes the clasp-closure devices implanted between January 2013 and 31 January 2020. MSA unit replacement sales were used to determine the rates. An institution initially purchases the devices as a set of four or five sizes, depending on the geography. This is to ensure the proper size is available as the size is determined intraoperatively for each patient. As the stock at the institution is replenished, it is assumed that a device has been implanted. The endpoint was based upon the time from implant to explant in months. All removals, regardless of reason, were included in the analysis. Incomplete data were apparent in both the MAUDE database and the Ethicon complaint database. In situations where the time to explant could not be calculated based on incomplete dates of implant or explant, the time to explant was imputed using the empirical distribution of records with complete information. Multiple imputation methods were employed across a total of *m* = 5 imputations. Results across the imputed datasets were then pooled into single estimates and were summarized as described in Schafer.^7^ Explant and erosion rates were calculated at yearly intervals, and the Kaplan-Meier estimator was used to estimate the cumulative 7-year rate of explant and erosion separately. Chi-square analyses were used to investigate the association between the rate of explant and the geographical region (USA or OUS), device size and year of implant.

## RESULTS

This publication is intended to inform based upon the currently available device design and sizes. This analysis includes patients from the geographies in which the clasp-closure MSA device was commercially available, beginning in 2013. It does not include the original device design that was secured by sutures nor the size 12-bead device. Neither of those device options are commercially available today. Table 1 summarizes the number of patients and centers from each geography and details on length of device implantation.

**Table 1:** Summary of clinical experience

Number of patients	
USA	24,070
OUS	3,709
Number of implanting centers	
USA	~350
OUS	~90
Median (Q1, Q3) implant duration (months)	19.6 (9.2, 33.3)
Number of patients by implant duration	
<1 year	8,836 (31.8%)
1–3 years	12,961 (46.7%)
>3–5 years	4,060 (14.6%)
>5 years	1,922 (6.9%)

### Explant rates

The total number of devices distributed during this time period is 27,779. The total number of devices that have been reported as having been removed during this time period is 609 (2.2%).

Table 2 summarizes the reasons for device removal and the corresponding frequencies and the mean time to removal. The most common reason for removal was dysphagia (47.9%) at a mean time of 10.9 months. The second most common reason for removal was persistent or recurrent GERD (20.5%) with a mean time to removal of 20.5 months. In 11.2% of cases, the device was removed for unknown/other reasons.

**Table 2:** Reason for device removal and mean time to removal

Reason for removal	Number of removals	Percentage of total removals	Mean time to removal, months (±SD)
Dysphagia	292	47.9	10.9 (11.9)
Persistent GERD	125	20.5	20.5 (13.0)
Erosion	27	4.4	25.0 (12.9)
Abdominal pain/pain	46	7.6	15.8 (14.3)
Discontinuous device†	17	2.8	33.7 (6.0)
Need for MRI	11	1.8	28.6 (13.2)
Vomiting	16	2.6	7.4 (8.2)
Gastroparesis	4	0.7	20.7 (18.5)
Device migration	3	0.5	12.6 (17.7)
Other/unknown	68	11.2	6.8 (6.4)
**Total removals**	**609**	**100.0**	**14.6 (13.4)**

^†^Discontinuous devices were the result of a manufacturing issue that resulted in a voluntary recall in 2018.

**Fig. 2:**
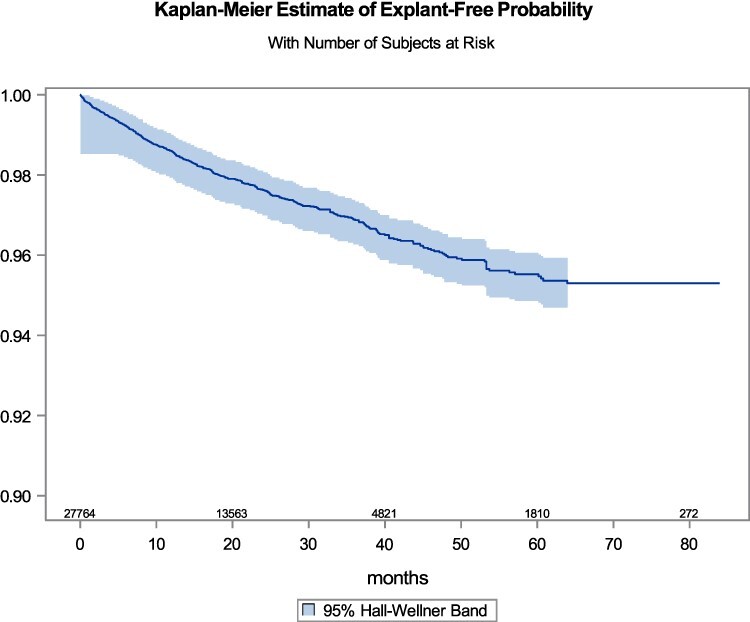
Kaplan Meier estimate of time to MSA removal

The overall 7-year cumulative risk of explant was 4.81% (95% CI: 4.31–5.36%). Figure 2 shows the estimated probability of the device remaining implanted as a function of time with the corresponding confidence band.

Device size was significantly related (Chi-square *P*-value < 0.0001) to the likelihood of an explant, with the smallest size having the highest explant rate. See Table 3 for a summary of the device size and percentage of removal rate.

**Table 3:** Device size and Removal rate

Device size	Removal rate (%)
13 beads	3.5
14 beads	2.4
15 beads	1.7
16 beads	1.5
17 beads	1.3

The rate of explant also varied by implant year, with implants placed in 2015 having the highest explant rate (5.8%), while the explant rates of implants placed in 2013, 2014 and 2016 were in the 3–4% range with rates ≤2.1% for more recent years. No clinically meaningful difference in the explant rates between the US sites (2.1%) and OUS sites (2.8%) was observed.

### Erosion rates

In the time period of the analysis, 27 devices were removed due to part of the device eroding through the esophageal wall and into the lumen. The cumulative risk of erosion at 7 years was 0.28% (95% CI: 0.17–0.46%).

Table 4 represents the breakout of device sizes and how each size contributed to the erosion totals. The 13-bead devices comprised ~30% of the total number of erosions. While the overall number of erosions is small and device size was not available in seven cases, it is notable that size may appear to have a correlation to erosion with the smaller sizes (13 and 14 beads) making up 65% of the total where the device size was known.

**Table 4:** Percentage of erosions relative to device size

Device size	Device size percentage of sales†	Total erosions	Percentage of erosions (*n*/27)	Percentage of removals (*n*/609)	Time to removal, months (SD)
13 beads	14.1	8	29.6	1.3	26.7 (10.7)
14 beads	25.1	5	18.5	0.8	27.5 (16.2)
15 beads	27.1	2	7.4	0.3	27.6 (16.4)
16 beads	20.6	2	7.4	0.3	28.9 (NA)
17 beads	13.1	3	11.1	0.5	7.5 (1.6)
Unknown	—	7	25.9	1.1	NA
**Total**	**100.0**	**27**	**100.0**	**4.4**	**25.0 (12.9)**

^†^Sales utilization data used as surrogate for number of devices implanted. NA = Not Applicable.

Given the low number of erosions overall, it was difficult to investigate other potential predictors associated with the risk of erosion.

### Sizing trend

In the feasibility and pivotal studies, the most common implanted size was the 14-bead device, with 55% (21/38) and 46% (46/100) of the implants, respectively.^8^

**Fig. 3:**
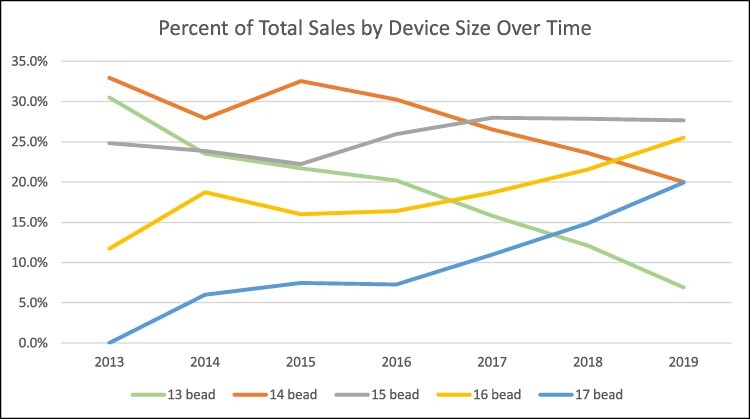
Sizing trend based upon device sales

Changes in clinical practice occurred over time, including the iterative sizing tool, are reflected in an increase in average device size from 14.2 ± 1.0 in 2013 to 15.3 ± 1.2 in 2019 (*P* < 0.001). Figure 3 shows the trend in device sizes sold over time. The 13- and 14-bead devices are trending downward, while the 16- and 17-bead devices are trending up. The 15-bead device remains relatively steady as the most implanted size, comprising ~27% of sales. Surgeons are finding that when using larger-sized devices, patients are experiencing adequate efficacy and less postoperative dysphagia.^9^^,^^10^^,^^24^

## DISCUSSION

The safety and efficacy of MSA have been well documented. To date, there have been over 55 peer-reviewed publications, including 2 RCTs, 8 meta-analyses, 30 cohort studies, 10 non-randomized comparative therapy outcome studies and 3 health economic studies. Three of these publications have focused entirely on the safety aspect of MSA.^5^^,^^11^^,^^12^

It is important to stress that this analysis includes only the current device design and available sizes, beginning in 2013. The objective is to inform on the safety profile of the currently available device. The publications on the removal and erosion rates referenced below do include all devices and designs, beginning with the feasibility study in 2007.

In 2015, Lipham *et al*. published the safety analysis of the first 1,048 patients implanted with MSA.^5^ This included the 38 and 100 patients implanted in the feasibility and pivotal studies, respectively. The rate of device removal was 3.4% (36/1048) and 64% (23/36) was for dysphagia. There was one erosion in this population for a rate of 0.1%. It is notable that in this early surgical experience, 17 of the 36 removals occurred in the first 3 months after implantation, with 94% (16/17) being removed for dysphagia. Surgeons have since developed protocols to address and manage early postop dysphagia.^6^

As a follow-up to Lipham *et al*., C. Daniel Smith *et al*. published their safety analysis on the first 3,283 implanted with MSA after FDA approval.^11^ The overall rate of removal was 2.7% (89/3283). Of these 89 removals, 58.4% were due to dysphagia (52/89), with 71% occurring within the first 12 months of implantation. The authors showed a downward trend over time for device removals. The rate of removal for erosion was 0.15% (5/3283), with most occurring between 12 and 24 months of implantation. Device removal for reasons other than erosion is typically done non-emergently in a laparoscopic procedure.

Device erosion is an important but rare complication seen with MSA. Alicuben *et al*. reported on the worldwide experience with MSA erosions.^12^ In the study period from 2007 to 2017, 9,453 devices were implanted with 29 erosions (0.3%) being reported through the manufacturer and/or the MAUDE database. Per the Kaplan-Meier method, the risk of erosion was 0.05% at 1 year of implantation, which increased to 0.3% after 4 years of implantation. Removal of an eroded device is typically managed non-emergently in a two-stage approach, with endoscopic removal of the visible beads in the esophageal lumen, followed 8–12 weeks later with the laparoscopic removal of the remaining device.

These publications show rates that are consistent with the current rates of removal, when calculated in the same fashion, with the removal rate at 2.2% (609/27,779) and the erosion rate slightly lower at 0.09% (27/27,779). This is significant because the number of implanted devices has greatly increased since the last safety-focused publication in 2017. When calculating rates using the Kaplan-Meier method that takes the patient-years of exposure of the device into account, the overall cumulative removal risk at 7 years was 4.81% (95% CI: 4.31–5.36%). The corresponding erosion risk at 7 years was 0.28% (95% CI: 0.17–0.46%). Device size was significantly associated with the risk of explant and appears to be related to the risk of erosion.

### Procedure evolution

As described by Bonavina *et al*., the initial implant procedure utilized a laparoscopic approach done under general anesthesia.^13^ The goal was to leave the native anatomy as intact as possible while placing the device. There was minimal disruption of the phrenoesophageal ligament, with just enough dissection to create the space to place the sizing tool and device around the esophagus at the level of the LES. If needed, a simple cruroplasty was done.

In 2020, Bonavina, in Ferrari *et al*.^14^ published on their single institution experience with MSA in 335 patients, beginning in 2007. This publication embodies the learnings over 12 years of implanting MSA. The primary focus of the publication is the outcomes of 124 patients who are between 6 and 12 years (median 9 years) post-MSA implant. In this group, the removal rate was 2.4%, with no erosions and no migrations. If considering the entire group of 335 patients, the removal rate was 9.2%, with majority of removals being associated with smaller device size (12 and 13 beads) and occurring prior to reaching 6 years of implantation.

As more experience was gained with MSA, it was noted anecdotally, that there appeared to be a higher-than-expected rate of hernia progression or recurrence and a return of GERD symptoms after minimal dissection to implant the device.^15^ More attention was given to the impact of the crural diaphragm and its contribution to the prevention of GERD. Historically, full crural dissection and hiatal hernia repair are standard as part of a fundoplication procedure.^16^ This two-sphincter hypothesis is supported by the pioneering work of Mittal *et al*. and is further explored by studies done by Shafik *et al*. These investigations show the crural diaphragm appears to contribute, along with the LES, in the control of GERD symptoms by its sphincteric-like action in response to diaphragmatic contraction and distention.^17^^,^^18^ Given this learning, in early 2017, the implant procedure was modified to include full crural dissection and a robust hiatal hernia repair. Tatum *et al*. retrospectively compared the results of patients who underwent MSA with minimal dissection (*n* = 90) and patients who underwent full crural dissection and hernia repair (*n* = 45).^15^ The results show a statistically significant reduction in the need for surgical repair for recurrent or persistent hiatal hernia in the full dissection group. In a similar study, Irribarra *et al*. concluded that an MSA procedure that included hiatal dissection, esophageal mobilization and crural closure provided the best outcome.^19^ These results have led to modifying the implant procedure to include the three tenets of anti-reflux surgery^20^:

- Reduce hiatal hernia and restore intrabdominal esophageal length
- Restore the angle of His
- Recreate/support the LES

Rona *et al*. and Buckley *et al*. published their experience comparing MSA outcomes in patients with hiatal hernias <3 cm and those patients with hiatal hernias >3 cm but repaired at the time of implant. Based on the similar results, they concluded the safety and efficacy of MSA implantation is independent of hernia size, as long as the hernia was addressed.^21^^,^^22^

Full description of minimal and full hiatal dissection is included in the supplementary materials.

### Postop management

With Nissen fundoplication, a patient is typically placed on a liquid diet immediately after surgery and then progressed to a puréed diet consisting of foods with pudding-like consistency, such as baby food, smoothies, etc., as tolerated. This diet progression continues until the patient is back to a regular diet, which is usually 4–6 weeks after surgery.^16^ It was discovered quickly that if a patient implanted with MSA was instructed to follow a typical post-Nissen diet, which was the early recommendation, the patient would likely experience significant dysphagia by 6–8 weeks. As the patient heals after MSA placement, a fibrous capsule forms around the device. The postoperative diet instructions are then quickly modified to instruct patients to begin eating semi-soft foods (fig newtons, crackers, yogurt, etc.) every 1–2 hours while awake. This is to ensure there is a bolus of food large enough to expand the device to create space while the encapsulation is occurring. This is presented to the patient as his/her ‘physical therapy’.

Some dysphagia is expected after MSA implantation, starting at 10–14 days post-implantation and peaking at 8–12 weeks. Setting appropriate expectations preoperatively is very important so patients are aware of the likelihood of early dysphagia and that it is a normal part of the healing process. Louie *et al*. addressed postoperative dysphagia and described the typical patterns of early and expected, persistent and late onset.^23^ Each is addressed differently depending on the timing and severity. Ayazi *et al*., in treating and following 380 patients implanted with MSA, proposed a treatment algorithm based on early (<8 weeks) versus late (>8 weeks) dysphagia.^9^ The authors found that early balloon dilation was effective in 21% of patients, as compared to 48.3% of patients, when done >8 weeks postop. Dilation was often accompanied by a short course of oral steroids.

As stated earlier, dysphagia was the most common reason for removal in our study as well as in previous investigations. Lipham *et al*. reported a removal due to dysphagia as 64% of the total removals; Smith *et al*., in the follow-up study, reported dysphagia as 58.4% of the total.^5^^,^^11^ In this study, including nearly 8 years of implant experience, the rate of removal for dysphagia was 47.9% of the total. There appears to be a slow decrease over time, which may reflect the changes in the surgical procedure and postop management. More research is needed to confirm and quantify this trend.

While Ethicon diligently pursues the information related to any device-related complication, it is important to recognize that this research also relies on the self-reporting nature of the MAUDE database. We certainly must acknowledge the potential for underreporting of device complications due to various factors, such as not understanding the importance of reporting a complication or even the process itself. It is possible that there may be devices removed in centers that were not formally trained on MSA implantation and there is a higher likelihood that such a removal may not be reported to the company or to MAUDE. We are only able to analyze and report on the information that has been received. Completeness of data is another limitation of this study, given the reliance on site-reported product complaints and the MAUDE database. This is apparent in the incomplete data information that was addressed through imputation as well as the reasonable number of ‘other/unknown’ reasons for removal, which may actually overlap or represent other specific categories of removal reasons. To counter this, one may look to those centers who have implanted a significant number of MSA devices and who also closely follow the outcomes of their patients. Ayazi *et al*., reported on their single center experience with MSA implantation and follow-up on 553 patients. There were no erosions reported, but 37 patients required device removal for a rate of 6.7%. Of the removals, over half (20/37) were due to dysphagia.^24^ Tatum *et al*. published their findings on follow-up of their single center experience with MSA. Of the 435 patients, 24 required device removal for a rate of 5.5%.^15^ This may be more in alignment with real-world experience, given the limitation of relying on reported information.

### Conclusion

Surgical technique and perioperative management play an important role in patient outcomes. Clinical practice changes since MSA has been incorporated into clinical use MSA are associated with improved outcomes and should be further characterized and disseminated. Full crural dissection with concomitant hiatal hernia repair should be incorporated into the procedure. Smaller device size is associated with increased explant and erosion rates when compared to larger-sized devices.

## CONFLICT OF INTEREST

All the authors are employed by Ethicon Inc.

## Supplementary Material

Figure_4_Supplementary_information_doab036

SUPPLEMENTARY_INFORMATION_no_figures_doab036
