# Supplementary material for: Evolution of a novel technology for gastroesophageal reflux disease: a safety perspective of magnetic sphincter augmentation
Source: Dis Esophagus. 2021 Jun 11;34(11):doab036. doi: 10.1093/dote/doab036 (PMC8597906; doi:10.1093/dote/doab036)
Supplement: Figure_4_Supplementary_information_doab036 [file figure_4_supplementary_information_doab036.pdf]

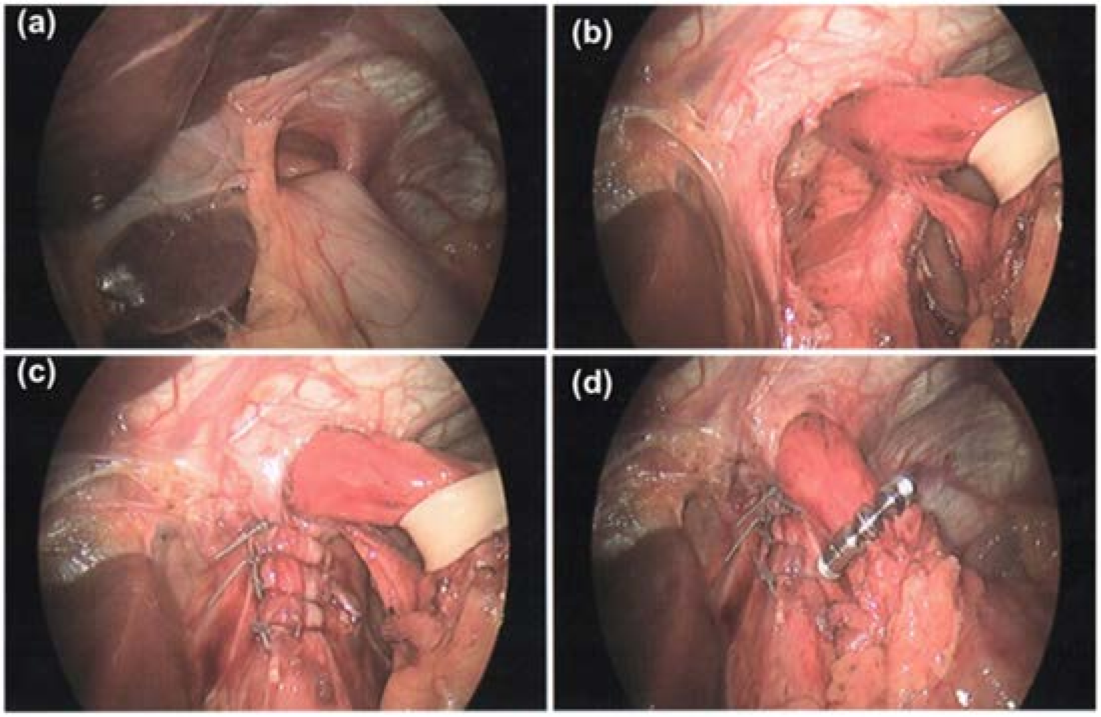

Fig. 4 Steps of hernia repair andmagnetic sphincter augmentation in a patientwith large paraesophageal hernia (Photo used with permission from Shahin Ayazi, MD)
