# Supplementary material for: Evolution of a novel technology for gastroesophageal reflux disease: a safety perspective of magnetic sphincter augmentation
Source: Dis Esophagus. 2021 Jun 11;34(11):doab036. doi: 10.1093/dote/doab036 (PMC8597906; doi:10.1093/dote/doab036)
Supplement: SUPPLEMENTARY_INFORMATION_no_figures_doab036 [file supplementary_information_no_figures_doab036.docx]

Supplementary Information

Full description of minimal dissection procedure [Bonavina 2008]:

- *The visceral peritoneum is divided at the gastroesophageal junction to expose the anterior esophagus. Sparing the hepatic branch of the vagus nerve, the retro-esophageal dissection begins along the right crus just above the point of decussation.*
- *The same dissection is performed along the left crus of the diaphragm. Gentle dissection posterior to the esophagus from right to left opens a retroesophageal window.*
- *A small tunnel is created between the posterior vagus nerve and the posterior esophageal wall and a ~10cm length of 6mm Penrose drain is placed to hold the space.*
- *The sizing tool is inserted and placed in this small tunnel between the vagus nerve and esophagus and pulled around the esophagus to determine the appropriate-sized device.*
- *Once selected, the device is placed into the abdominal cavity, positioned around the esophagus, ensuring the device is placed between the posterior vagus nerve and posterior esophagus through the created tunnel. This placement is designed to tether the device at the gastroesophageal junction until the device has encapsulated and is stable.*
- *The sutures on each end of the device are secured with a small Ti-knot crimping tube (LSI solutions, Victor NY, USA).*
- *In this initial group of patients, the median operative time was 40 minutes (range 19 – 104).*

Full description of full dissection procedure American Foregut Society website:

<https://www.americanforegutsociety.org/wp-content/uploads/2019/10/AFS-LINX-Final.pdf>

*PART 1: HIATUS*

- *See Figure 4a. for example of large paraesophageal hernia prior to repair.*
- *The phrenoesophageal membrane is circumferentially dissected, allowing entrance into the mediastinum. This dissection may entail incision of the gastrohepatic ligament as well as the hepatic branch of the vagus nerve.*
- *The opening of the phrenoesophageal membrane allows proper evaluation of the gastroesophageal junction, visualization of the anterior and posterior vagus nerves to ensure they are left intact and the ability to assess intra-abdominal esophageal length.*
- *The careful dissection should extend deep into the mediastinum, along the avascular plane adjacent to the esophagus, with attention to the vagal nerves. Blunt dissection is often effective and can be supplemented with cautery to control minor bleeding. If extensive bleeding occurs, stop and re-evaluate the dissection plane.*
- *Dissection is continued to ensure a minimum of 2cm of intra-abdominal esophagus after crural repair to allow safe MSA implantation. More length is better if one can safely achieve it.*
- *Upper endoscopy can be helpful at this point in the procedure to identify proper anatomical structures.*
- *In the case of a paraesophageal hernia, the hernia sac must be carefully dissected and reduced. Continued caudal retraction of the hernia sac creates tension at the level of the dissection plane. In some cases, a large amount of stomach can be in the mediastinum, making the dissection much more difficult due to poor planes and visibility.*
  - *Once the mediastinal dissection is complete, regardless of hernia type or size, the available intra-abdominal esophageal length must be assessed to ensure adequate length for safe MSA placement. (See Figure 4b.) The hiatus is then closed by approximating the crural muscles with suture, typically posteriorly and sometimes anteriorly as well, depending on the size of the repair. The correct approximation of the crura to the wall of the esophagus is thought to be critical in reconstructing the GERD barrier as one part of the two-sphincter hypothesis.*
  - *The use of mesh and the suturing method are at the discretion of the surgeon.*

*PART 2: LOWER ESOPHAGEAL SPHINCTER AND MSA IMPLANTATION*

- *After the hiatus is closed and ensuring there is at least 2cm of intra-abdominal esophagus, a small window is created between the esophagus and posterior vagus nerve at the level of the LES. This small opening is preserved by placing a Penrose drain to hold the space. (See Figure 4c.)*
- *The sizing tool is placed through the most lateral right port and through the opening between the posterior vagus nerve and esophagus, utilizing the Penrose tube.*
- *Confirm that there is nothing in the esophagus (nasogastric tube, bougie, etc.) prior to deploying the sizing tool.*
- *The correct size is determined by incrementally tightening the flexible tip portion of the sizing tool around the esophagus until there is contact circumferentially but no compression of tissue. The corresponding device size can be read off the scale on the shaft of the tool. This should be repeated 3 times to ensure consistency. The sizing tool can be tightened until the magnetic tip releases, noting the size in which it “pops off”. Adding two or three to this figure should correlate with the initial size determination. If in between, it is advisable to size up.*
- *The device is opened and placed down a trocar (Must be >5mm in diameter). The device is then placed around the esophagus, again using the Penrose drain to ensure it is placed between the posterior vagus nerve and the esophagus. The two halves of the clasp are brought together and closed to secure the device and the sutures removed. (See Figure 4d.)*
- *Mean op time (Tatum full dissection) was 69.3 +/-27 minutes.*
